# The dRM-Index: Applicability of the Differential Respiratory-to-Muscular Training Load Index in Young Female Soccer Players

**DOI:** 10.5114/jhk/211330

**Published:** 2026-04-02

**Authors:** Oihan Esnal-Arrizabalaga, Ibai Garcia-Tabar, Asier Gonzalez-Artetxe, Asier Los Arcos

**Affiliations:** 1 Department of Physical Education and Sport, Faculty of Education and Sport, University of the Basque Country UPV/EHU, Vitoria-Gasteiz, Araba, Spain.; 2 Department of Strength and Conditioning, Sociedad Deportiva Eibar, Eibar, Gipuzkoa, Spain.; 3 Society, Sports, and Exercise Research Group (GIKAFIT), University of the Basque Country UPV/EHU, Vitoria-Gasteiz, Araba, Spain.; 4 Physical Activity, Exercise, and Health Group, Bioaraba Health Research Institute, Vitoria-Gasteiz, Araba, Spain.

**Keywords:** women’s sport, team sports, internal load, perceived effort, physical fitness

## Abstract

This study described the differential respiratory and muscular perceived training loads (RPEres-TL and RPEmus-TL) within and between sessions, and investigated the associations between neuromuscular performance and the dRM-Index (i.e., the ratio of differential respiratory and muscular perceived training loads: RPEres-TL divided by RPEmus-TL) among youth female soccer players. Thirteen outfield players (age: 17 ± 1 years) of the third team of a Women’s Soccer Spanish First Division Club completed the 14-week study period. Neuromuscular performance tests (countermovement jump [CMJ], CMJ arm swing, single leg CMJ, and 30-m sprint tests) were conducted before (T1) and after (T2) the study period. Players improved neuromuscular performance (p < 0.05; Cohen’s d < 1.29; small-to-large) from T1 to T2. RPEres-TL was greater than RPEmus-TL for training sessions performed four (M−4: p < 0.001; Cohen’s d = −0.61; moderate) and one (M−1: p = 0.01; Cohen’s d = −0.37; small) days before the match. Baseline sprint performance showed large correlations with the dRM-Index during M−2 (p < 0.05; r < 0.59), M−1 (p < 0.05; r < 0.60) and matches (p < 0.05, r < 0.66). Differential RPE allows the analysis of the TL distribution during the week providing additional insights into the training workload. Baseline sprint performance can be used to forecast the differed heterogeneous individual cardiovascular and neuromuscular responses to the sessions. This initial study on the dRM-Index offers a novel and practical approach to individualize and optimize the training process of youth female soccer players.

## Introduction

Monitoring the players’ training load (TL) is commonplace among soccer teams (Impellizzeri et al., 2019; Savolainen et al., 2025). Monitoring of the TL is fundamental to prescribe and control the desired training stimuli, optimize recovery, avoid overtraining, reduce injury risk, and enhance physical preparedness to compete (Douchet et al., 2024; Drew and Finch, 2016; Impellizzeri et al., 2019; Jaspers et al., 2017; Silva et al., 2018). Among the methods for quantifying the TL, the global rating of perceived exertion (RPE) stands out for its practical advantages. Its simplicity (Borg, 1982), the lack of requirement for qualified professionals (Alexiou and Coutts, 2008), cost-effectiveness (Borg, 1982), and validation as a non-invasive indicator of the internal TL (Foster et al., 2001; McLaren et al., 2018) makes it particularly noteworthy and most of the time applicable in real-world settings. These advantages are even more relevant in youth athletes, given the constraints on human and material resources, which limit the use of other more expensive methods (e.g., heart rate and global positioning systems) requiring qualified personnel and more sophisticated time-consuming data extraction procedures (Dudley et al., 2023).

Positive and robust associations have been observed among RPE-derived TL, heart rate and external TL variables during soccer training and matches (McLaren et al., 2018). However, it is considered that a single-item measure of effort (i.e., global RPE) is insufficient to capture the whole range of perceptual sensations during exercise training (Hutchinson and Tenenbaum, 2006). A differential RPE, which involves the deconstruction of the RPE gestalt, extends internal load measurement by recording data that provide additional worthwhile information to TL monitoring procedures (McLaren et al., 2016, 2018). This deconstruction allows the assessment of central (i.e., breathlessness) and peripheral (i.e., leg-muscle exertion) loads (Borg et al., 2010; Ekblom and Golobarg, 1971; Pandolf, 1982) during training and matches by the recording of the distinct respiratory (RPEres) and muscular (RPEmus) RPEs. These differentiated measures have been satisfactorily applied to quantify the perceived TL (i.e., RPEres and RPEmus × volume in minutes [RPEres-TL and RPEmus-TL]) in male soccer players (Azcárate et al., 2018; Gil-Rey et al., 2015). In contrast to male counterparts, Wright et al. (2020) reported unclear differences between RPEres and RPEmus for soccer-specific training in youth female players. Despite insights gained, limited knowledge exists regarding the differential RPE in both youth male (Dudley et al., 2023; Gil-Rey et al., 2015) and female (Wright et al., 2020) players. Considering the physiological sex differences in muscle properties and composition (Ansdell et al., 2020; Tortu et al., 2024), together with previous unclear results (Wright et al., 2020), further exploration of the differential RPE in youth female soccer players is deemed necessary.

Martinho et al. (2024) suggested that neuromuscular performance predicted the global RPE in youth male soccer players. That study found that faster players perceived higher exertion levels during training sessions over a four-week preseason period (Martinho et al., 2024). This finding offers valuable insight for coaches to tailor training periodization based on each player’s neuromuscular capability in male youth soccer. In this sense, the differential respiratory and muscular RPE-TLs, as well as their ratio (dRM-Index: RPEres-TL divided by RPEmus-TL), may provide additional depth to training monitoring (McLaren et al., 2018). The dRM-Index, as a single metric, captures both neuromuscular and cardiovascular profiles, allowing the identification of differentiated internal training responses such as “speed-muscular” (dRM-Index < 1), “endurance-respiratory” (dRM-Index > 1), or “hybrid” (dRM-Index ≈ 1) trends (Sandford et al., 2021) at both team and individual levels. However, to the best of our knowledge, no study has examined whether the differential RPE-TL or the dRM-Index are associated with neuromuscular performance in female soccer players.

Therefore, this study aimed to (1) quantify and compare the RPEres-TL and the RPEmus-TL within and between training sessions and matches, and (2) assess the associations between neuromuscular performance and the dRM-Index in youth female soccer players during a 14-week period. Understanding respiratory and muscular TL distribution and accumulation, and their relationships with players’ neuromuscular performance, may provide insights for optimizing the training process in female soccer. It was hypothesized that (1) the RPEres-TL would be greater than the RPEmus-TL across training sessions and matches; (2) the TL would progressively decrease throughout the training week in preparation for the match, and increase again on the matchday to reflect the game’s higher demands; and (3) players’ neuromuscular profiles would be reflected in their training response, as captured by the dRM-Index.

## Methods

### Participants

Eighteen Basque female players (age: 17 ± 1 years, body height: 1.65 ± 0.05 m, body mass: 56.7 ± 6.37 kg; body fat content: 10.4 ± 1.63%; playing experience: 10 ± 2 years) of the third team of a Women’s Soccer Spanish First Division Club participated in the study. As players received structured and periodized training, developed their soccer skills in a professional academy environment, and competed in the first autonomous division (i.e., the Basque League), they were classified in Tier 3: Highly Trained/National Level athletes of the Participant Classification Framework (McKay et al., 2022). Two players faced long-term injuries (more than four weeks), while two others missed the first or the second testing session. Additionally, one player trained regularly with the inferior team. The two goalkeepers were excluded due to their distinct training and match workloads compared to outfielders. Hence, 13 players (5 defenders, 5 midfielders, and 3 forwards) were finally included in the study. Age, body height, body mass, body fat content and playing experience of the participants were 17 ± 2 years, 1.65 ± 0.05 m, 56.6 ± 6.83 kg, 10.5 ± 1.74% and 10 ± 2 years, respectively.

Players, their parents/guardians, coaches, and the club management were fully informed about the purpose and procedures of the study. Participants, and when appropriate their parents or legal guardians, acknowledged voluntary participation through written informed consent. The study followed the ethical principles for medical research involving human subjects of the World Medical Association (Declaration of Helsinki, 2013) and was approved beforehand by the Ethics Committee for Research involving Human Beings (GIEB in Basque) of the University of the Basque Country UPV/EHU, Leioa, Bizkaia, Spain (protocol code: M10_2021_328; approval date: 25 November 2021).

### Design and Procedures

A longitudinal observational study aiming to describe and analyze the differential RPE-TL and dRM-Index association with neuromuscular performance was performed. This study was conducted during 14 weeks from August to November, including testing 1 (T1) during the first week (last week of the preseason), 12 competitive weeks, and testing 2 (T2) during the 13^th^ week of the studied period. Testing sessions included the evaluation of vertical jump performance (countermovement jump [CMJ], arm swing CMJ [CMJAS], along with single leg dominant CMJ [CMJD] and single leg non-dominant CMJ [CMJnD]), and sprint performance (10-, 20- and 30-m distances). During the study, individual players’ differentiated RPEs (Los Arcos et al., 2015; Romero-Moraleda et al., 2023; Wright et al., 2020) for the entire training sessions and matches (Foster et al., 2001) were recorded.

### Training Schedule

**Figure 1:**
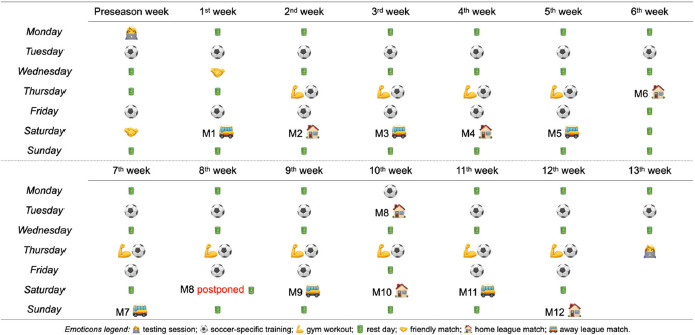
Training and matches’ schedule of the soccer team during the 14-week study period.

Players habitually trained three times per week (Tuesday [four days before the matchday: M−4], Thursday [M−2], and Friday [M−1]) and played an official match (Saturday [M]) during the competitive period after T1 week and before T2 week (Figure 1). The first session of the week (M−4) consisted of small-sided games (SSGs) with and without floaters and mini-goals, and large-sided games (LSGs) with mini-goals; during the M−2 gym workout, SSGs with goalkeepers, and LSGs with goalkeepers and offensive actions without defenders were carried out; and M−1 consisted of collective passing drills, build-up, finalizations, and set pieces. All training sessions were performed on an artificial turf field where all the male and female teams of the academy habitually trained. In total, the team carried out 36 training sessions, and played two friendly and 12 official matches during the study period (Figure 1).

### Measurements

Testing sessions were integrated into weekly training schedules according to the staff of the team. Tests were performed in the same order, after one day of minimal physical activity at 5.30 p.m., in the club’s indoor gymnasium. Participants were familiarized with the testing procedures during the warm-up of the previous training session. Neuromuscular performance tests were conducted after a standardized 15-min warm-up period that included low-intensity running, several acceleration runs, and several jumps and sprints. Participants were vigorously encouraged to perform maximally in every test. Tests were supervised by the same experienced assessors who were proficient in the test protocols, and also the staff of the team to ensure players’ commitment.

#### Vertical Jumping Tests

Four different jump tests were carried out in the following order: CMJ, CMJAS, CMJD and CMJnD. All tests were performed according to the procedures proposed by Bosco et al. (1983) and recorded by a contact platform (Microgate OptoJump Next, Bolzano, Italy). The CMJ protocol was as follows: players had to perform the jump with the hands fixed on the hips; the jump was required to begin from an extended leg position going down to 90° knee flexion, immediately followed by a subsequent concentric action for maximal height; additionally, players were instructed to land on the contact platform in a position similar to that of the take-off. Players performed three attempts of each test with a minimum 20-s recovery between each repetition. The highest jumping height for each of the different jumps was retained for further analysis. Test reliability (intraclass correlation coefficients [ICCs]) was > 0.94 and coefficients of variation (CVs) < 6.0%.

#### 30-m Linear Running Sprint Test

The sprint test consisted of three maximal sprints of 30 m, with a rest interval of 3 min in between. Running time was recorded with accuracy of 0.001 s using photocell gates (Microgate Polifemo, Bolzano, Italy) placed 0.50 m above the ground. The sprint was self-initiated from a standing start, 0.50 m behind the start line. The time was automatically activated when the participant passed the first gate at the 0-m mark, and split times were recorded at 10 (S10), 20 (S20) and 30 (S30) m. The best time at each distance was selected for further analysis. Test reliability (ICCs) was > 0.85% and CVs < 2.0%.

### Training Load Quantification

Internal TL data collection started after T1 (i.e., 28^th^ August), and finished with the last training session before T2 (i.e., 21^st^ November). Players were familiarized with the TL registration procedures during the four weeks before the T1. The perceived TL method was utilized to quantify the TL (Foster et al., 2001). Ten minutes after each training session and match (Azcárate et al., 2018; Los Arcos et al., 2015), players were asked to rate their perceived exertion (RPE) separately for respiratory (RPEres) and leg musculature (RPEmus) efforts (Azcárate et al., 2018; Los Arcos et al., 2015; Wright et al., 2020) using the Foster’s 0–10 scale (Foster et al., 2001). The same experienced investigator (i.e., the strength and conditioning coach) asked participants for their RPE and conducted the recording procedure on all the occasions. This investigator was proficient in this RPE recording procedure. Players were allowed to mark decimals plus sign alongside the integer value. The TL was calculated multiplying the RPE value by the duration of the training session or the match (Foster et al., 2001): RPEres- TL and RPEmus-TL. The duration of training sessions was recorded for each player from the start to the end of the session. The duration of matches, excluding the warm-up and in-between half-time rests, was also recorded for each player. The mean and total accumulated RPEres-TL and RPEmus-TL values of each player for training sessions (M−4, M−2, and M−1) and matches were calculated for descriptive analyses. The dRM-Index (i.e., the ratio between RPEres-TL and RPEmus-TL) for training sessions (M−4, M−2, M−1) and matches was calculated and considered for further descriptive and regression analyses. Only those players available to train with the team for the entire week (Azcárate et al., 2018; Los Arcos et al., 2017) were considered so as not to disturb the team’s mean descriptive results. Hence, three hundred and fifty-three observations were registered, 104 per each type of training (5–7 per player) and 41 match observations (2–6 per player).

### Statistical Analysis

Descriptive results are presented as means ± standard deviations (SDs) for neuromuscular performance values, and as means ± 95% confidence intervals (CIs) for RPE-TL data. Data were analyzed using parametric statistics following confirmation of normality using the Shapiro-Wilk test for neuromuscular performance and the Kolmogorov-Smirnov test for RPE-TL data. Homoscedasticity was checked by the Levene’s test. Paired *t*-tests were used to compare neuromuscular performance from T1 to T2. A linear mixed model (with Bonferroni post hoc tests) was used to compare total RPEres-TL and RPEmus-TL, weekly RPEres-TL and RPEmus-TL, and intra-session RPEres-TL and RPEmus-TL; and to assess the periodization of daily session RPEres-TL, RPEmus and dRM-Index within the training week (Newans et al., 2022). The TL type (RPEres-TL and RPEmus-TL) was included as fixed effect and individual players were random effects for the *first model*. The weekday/type of the session (M−4, M−2, M−1, and M) was included as fixed effect and individual players were random effects for the *second model*. Cohen’s *d* effect sizes with 95% CIs were used to interpret the magnitude of the differences. Therefore, Cohen’s *d* < 0.20, 0.20–0.50, 0.50–0.80 and ≥ 0.80 were considered *trivial, small, moderate* and *large*, respectively (Cohen, 1988). The CV was used to calculate between players RPEres-TL and RPEmus-TL variability for the total accumulated TL during the study period and the weekly TL. Pearson’s product-moment correlation coefficients (r) were used to assess the direction and magnitude of the linear relationships between players’ neuromuscular performance and the dRM-Index. The accuracy of each linear regression was evaluated using the 95% CIs. The magnitude of correlations was interpreted according to the thresholds previously recommended (Hopkins et al., 2009). Evaluation of Cook’s distance revealed a minimal influence of the individual data points on the correlation magnitude. A *p* value < 0.05 was considered significant for analyses not requiring post hoc adjustments. Analyses were performed using SPSS Statistics version 22.0 for Windows (IBM, Armonk, NY, USA).

## Results

Neuromuscular performance test results are reported in Table 1. Players improved (*p* < 0.05; Cohen’s *d* < 1.29; *small*-to-*large*) their CMJ, CMJAS, S10, S20 and S30 performances from T1 to T2.

A significant effect of the TL type (*p* = 0.02) was observed for the total accumulated team mean RPE-TL. Total RPEres-TL (15230 [14006; 16455] arbitrary units [AUs]; CV = 12.5%) was greater (Cohen’s *d* = −0.80 [−1.93; 0.33], *large*) than RPEmus-TL (13528 [12303; 14753] AUs; CV = 17.2%). The total team mean dRM-Index was 1.15 [1.05; 1.26]. Significant main effect of the TL type (*p* = 0.07; Cohen’s *d* = −0.38 [−1.00; 0.25], *small*) was not observed for the weekly RPE-TL (RPEres-TL = 1525 [1391; 1660] AUs, CV = 21.5%; RPEmus-TL = 1398 [1263; 1532] AUs, CV = 24.7%).

**Table 1:** Physical testing results (means ± standard deviations) before (T1) and after (T2) the study period (*n* = 13).

	T1	T2	∆ (%) [95%, CI]	*p*	Cohen’s *d* effect size
CMJ (cm)	28.1 ± 4.77	29.5 ± 7.46*	5.47 [1.98; 9.09]	0.01	0.22 [−0.87; 1.31]
CMJAS (cm)	32.5 ± 5.52	33.6 ± 5.65*	3.24 [0.41; 6.16]	0.04	0.20 [−0.89; 1.29]
CMJD (cm)	16.2 ± 2.75	17.1 ± 3.05	5.33 [−0.52; 11.52]	0.07	0.31 [−0.78; 1.40]
CMJnD (cm)	16.1 ± 2.83	17.3 ± 3.06	7.23 [−0.65; 15.74]	0.07	0.41 [−0.69; 1.51]
S10 (s)	1.94 ± 0.09	1.83 ± 0.08#	−5.86 [−6.77; −4.95]	< 0.001	−1.29 [−2.49; −0.10]
S20 (s)	3.41 ± 0.15	3.29 ± 0.14#	−3.60 [−4.53; −2.67]	< 0.001	−0.83 [−1.96; 0.31]
S30 (s)	4.85 ± 0.22	4.71 ± 0.21#	−2.88 [−3.81; −1.93]	< 0.001	−0.65 [−1.77; 0.47]

Abbreviations: Δ, change from T1 to T2; CI, confidence intervals; CMJ, countermovement jump; CMJAS, arm swing CMJ; CMJD, single leg dominant CMJ; CMJnD, single leg non-dominant CMJ; S10, sprint time at 10 m; S20, sprint time at 20 m; S30, sprint time at 30 m. Note: *, significantly (p < 0.05) higher than T1; #, significantly (p < 0.05) faster than T1.

**Figure 2:**
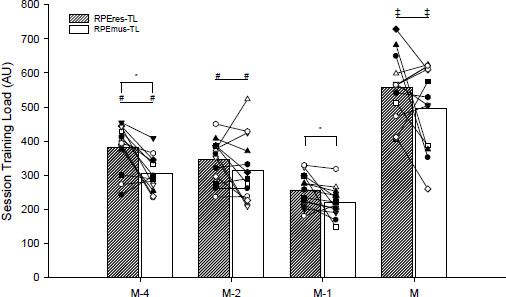
Distribution of perceived respiratory (RPEres-TL) and muscular (RPEmus-TL) training loads along the week during the 12-week competitive period (*n* = 13). Columns indicate the mean of the team for both the RPEres-TL and the RPEmus-TL for each training or match. Symbols, linked with black solid lines, indicate the individual means of each of the players for both the RPEres-TL and the RPEmus-TL for each training or match. M−4, M−2, M−1 are the training sessions performed on 4, 2 and 1 days before the match (M), respectively. Note: *, significant intra-session differences at the p < 0.05 level; #, significantly different from M−1 session at the p < 0.001 level; ‡, significantly different from the rest of the training sessions at the p < 0.001 level

Figure 2 shows the mean for the team (columns) and the individual mean for each player (symbols) for both the RPEres-TL and the RPEmus-TL during the week for each type of the session. A significant main effect of the type of the session (*p* < 0.001) was observed for the RPEres-TL, being greater in matches than in training: M−4 (*p* < 0.001; Cohen’s *d* = −1.32 [−1.79; −0.86], *large*), M−2 (*p* < 0.001; Cohen’s *d* = −1.67 [−2.16; −1.18], *large*), and M−1 (*p* < 0.001; Cohen’s *d* = −2.56 [−3.13; −1.99], *large*). Respiratory loads were also greater on M−4 (*p* < 0.001; Cohen’s *d* = −1.00 [−1.45; −0.55], *large*) and M−2 (*p* < 0.001; Cohen’s *d* = −0.83 [−1.27; −0.39], *large*) compared to M−1. No significant differences were found between M−4 and M−2 (*p* = 0.44; Cohen’s *d* = −0.24 [−0.66; 0.18], *small*) for the RPEres-TL. A significant main effect of the type of the session (*p* < 0.001) was also apparent for the RPEmus-TL. Muscular loads were greater in matches than in training: M−4 (*p* < 0.001; Cohen’s *d* = −1.35 [−1.81; −0.88], *large*), M−2 (*p* < 0.001; Cohen’s *d* = −1.03 [−1.48; −0.59], *large*), and M−1 (*p* < 0.001; Cohen’s *d* = −1.94 [−2.45; −1.43], *large*). The RPEmus-TL values were also greater on M−4 (*p* < 0.001; Cohen’s *d* = −0.71 [−1.14; −0.28], *moderate*) and M−2 (*p* < 0.001; Cohen’s *d* = −0.68 [−1.11; −0.24], *moderate*) compared to M−1. No significant differences were found between M−4 and M−2 (*p* = 1.00; Cohen’s *d* = 0.14 [−0.28; 0.56], *trivial*) for the RPEmus-TL.

Intra-sessions’ analyses showed the RPEres-TL to be greater than the RPEmus-TL for M−4 (*p* < 0.001; Cohen’s *d* = −0.61 [−1.04; −0.17], *moderate*) and M−1 (*p* = 0.01, Cohen’s *d* = −0.37 [−0.79; 0.06], *small*) training sessions (Figure 2). No significant differences were found for the M−2 training session (*p* = 0.22, Cohen’s *d* = −0.17 [−0.60; 0.25], *trivial*) nor the match (*p* = 0.13, Cohen’s *d* = −0.31 [−0.74; 0.11], *small*).

**Figure 3:**
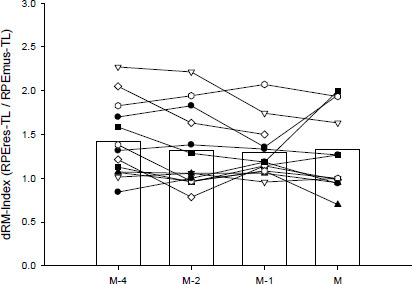
Distribution of the dRM-Index (i.e., the ratio between differential respiratory [RPEres-TL] and the muscular [RPEmus-TL] training loads) during the week in the 12-week competitive period (*n* = 13). Columns indicate the mean of the team for each training or match. Symbols, linked with black solid lines, indicate the individual means of ach of the players for each training or match. M−4, M−2, M−1 are the training sessions performed 4, 2 and 1 days before the match (M), respectively

Figure 3 presents the mean for the team (columns) and the individual mean for each player (symbols) for the dRM-Index during the week for each type of the session. Significant main effect of the weekday (*p* = 0.50) was not observed for the dRM-Index (M−4 = 1.42 [1.17; 1.66]; M−2 = 1.32 [1.07; 1.56]; M−1 = 1.30 [1.05; 1.54]; M = 1.33 [1.06; 1.60]): M−4 vs. M−2 (Cohen’s *d* = −0.14 [−0.56; 0.28], *trivial*), M−4 vs. M−1 (Cohen’s *d* = −0.18 [−0.60; 0.24], *trivial*), M−4 vs. M (*d* = −0.24 [−0.66; 0.18], *small*), M−2 vs. M−1 (*d* = −0.03 [−0.45; 0.39], *trivial*), M−2 vs. M (*d* = −0.09 [−0.51; 0.34], *trivial*) and M−1 vs. M (*d* = −0.06 [−0.48; 0.36], *trivial*).

**Figure 4:**
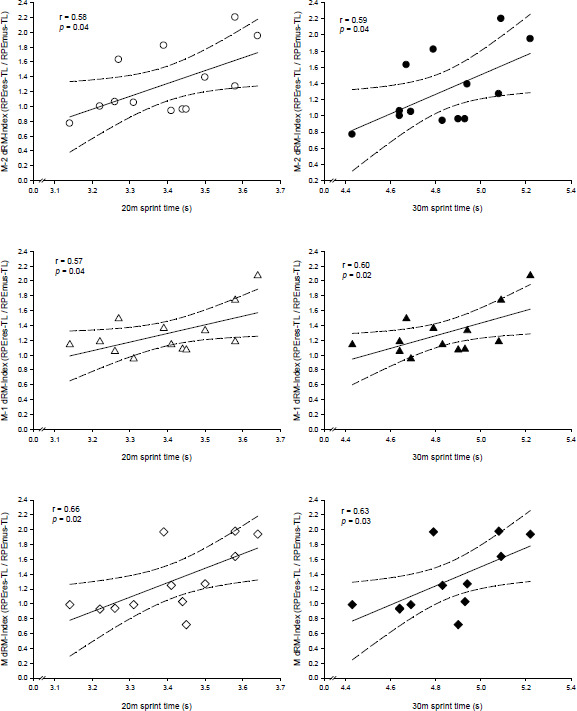
Individual data points and linear relationships between the session dRM-Index (i.e., the ratio between differential respiratory [RPEres-TL] and muscular [RPEmus-TL] training loads) for M−2 (upper panel), M−1 (middle panel) and M (lower panel) with 20- (left panel) and 30-m (right panel) sprint performance. M−2 and M−1 are training sessions performed 2 and 1 days before the match (M), respectively. Solid lines: linear regressions; dashed lines: 95% confidence intervals

Sprint performance at T1 was *largely* related to the dRM-Index of M−2, M−1, and matches (Figure 4). Total dRM-Index and the dRM-Index of M−4 did not significantly correlate with sprint performance (*p* > 0.05). No significant correlations were found between vertical jump performances and the total, training, or match session dRM-Index (*p* > 0.05).

## Discussion

This study aimed to assess and compare respiratory (RPEres) and muscular (RPEmus) perceived exertion training loads (RPEres-TL and RPEmus-TL) between and within training sessions (M−4, M−2, M−1) and matches, and assess the association between neuromuscular performance and the ratio between RPEres and RPEmus TLs (i.e., dRM-Index) in youth female soccer players. The hypotheses were partially confirmed: (1) the TL decreased throughout the training week (M−4 and M−2 > M−1) reaching its lowest point in the M−1 session, followed by a discernible increase for the match, the day with the highest TL; (2) the RPEres-TL was greater than the RPEmus-TL for soccer-specific training sessions (M−4 and M−1), while no differences were found for the sessions including strength training (M−2) or competitive matches; and (3) *large* positive associations were found between sprint performance and training response as captured by the dRM-Index.

In the realm of soccer teams, the systematic quantification and assessment of the accumulated TL are of importance. High TLs and congested competition may cause residual fatigue degrading physical preparedness to compete (Jaspers et al., 2018). In our study, the total accumulated RPEres-TL during the in-season period was greater than the RPEmus-TL (*p* = 0.02, Cohen’s *d* = −0.80, *large*) and a similar trend (*p* = 0.07; Cohen’s *d* = −0.38, *small*) was found for the weekly workload. This finding contrasts with research on junior non-elite male players, where a greater accumulated RPEmus-TL was noted compared to the RPEres-TL (Gil-Rey et al., 2015). These contrasting results might be related to the sex-specific skeletal muscle properties and composition characteristics (Ansdell et al., 2020). Female athletes usually demonstrate heightened lipid utilization and a greater tendency for muscle glycogen preservation at a given exercise intensity (Tarnopolsky et al., 1990). This might be the reason why female players attained the lactate threshold at a higher percentage of their maximal aerobic speed compared to their male counterparts (Garcia-Tabar et al., 2022; Skalski et al., 2024). Thus, our findings, together with the contrasting results previously reported in young males (Gil-Rey et al., 2015), suggest that for soccer training female players might show a greater oxidative and cardiovascular strain (RPEres-TL > RPEmus-TL), while males might show a higher neuromuscular response (RPEres-TL < RPEmus-TL). Further studies are required to corroborate these initial findings. However, these preliminary results indicate the potential utility of the total dRM-Index on-field recording as a practical approach to assess and monitor neuromuscular and cardiovascular responses in female soccer during training.

The perceived TL, as measured by both the RPEres-TL and the RPEmus-TL, tended to decrease progressively throughout the week, reaching its lowest point in the M−1 session. Subsequently, there was a discernible increase in the TL for the match, marking the highest TL for the entire week (Figure 2). Our results are in line with the previous investigations in youth male soccer (Whitworth-Turner et al., 2019). Intra-session comparisons, on the other hand, revealed the RPEres-TL to be greater than the RPEmus-TL for soccer-specific training sessions (Figure 2). In contrast, Wright et al. (2020) found *trivial* differences between the RPEres and the RPEmus in these types of training sessions for younger (15 ± 1 years) female English players. These contradictory results could be due to the different maturation state of players (Martinho et al., 2022), U15 vs. U17, and the selection and implementation of the soccer-specific training drills (de Dios-Álvarez et al., 2022). It is well known that the manipulation of the relevant features of the game during training influences the physical and physiological responses to the session (de Dios-Álvarez et al., 2022; Los Arcos et al., 2025). The combination of strength exercises with soccer-specific tasks (M−2), however, reduced the difference between respiratory and muscular loads, indicating similar central and peripheral responses to the session (Figure 2). This aligns with previous outcomes where sessions oriented to specific neuromuscular training resulted in a higher RPEmus compared to the RPEres in youth female players (Wright et al., 2020). Thus, to obtain a similar respiratory and muscular response during the training session, the inclusion of specific strength exercises in addition to soccer-specific training seems an effective training strategy in youth female soccer players. In agreement with Wright et al. (2020), where *trivial* differences were found in U15 players, in this study no significant differences were found between the RPEres-TL and the RPEmus-TL during the competitive matches. These results suggest that, similarly to the inclusion of strength-type exercises in soccer training, the competitive match reduces the difference between the RPEres-TL and the RPEmus-TL in youth female players. This indicates that matches lead to a higher relative neuromuscular effort (RPEmus-TL relative to RPEres-TL, i.e., reduced dRM-Index) in comparison to soccer-specific training sessions. These results support the additional worthwhile information of a differential training and match RPE in the TL quantification of youth female soccer players, allowing a practical, but more detailed, training periodization. Nevertheless, further studies are required to clarify the consequences of the nature of soccer-specific training sessions in comparison to matches on youth female soccer.

There are few studies describing the relationship between the accumulated global RPE-TL and neuromuscular performance in youth male (Dudley et al., 2023) and female (Gonçalves et al., 2021) players, and the results are contradictory. In this regard, the differentiated RPE-TL might provide further insights (McLaren et al., 2018) into the clarification of these inconsistent associations. Similarly to the concept of the anaerobic speed reserve, the dRM-Index might account for the different “speed-muscular” (dRM-Index < 1), “endurance-respiratory” (dRM-Index > 1) or “hybrid” (dRM-Index ≈ 1) training responses depending on the players’ profile or characteristics (Sandford et al., 2021), such as fiber typology. Nevertheless, to the best of our knowledge, this is the first study describing the dRM-Index during the week and its relationship with neuromuscular performance in youth female soccer players. Despite the significant and substantial differences in the RPEres-TL and the RPEmus-TL between training and competition (Figure 2), the dRM-Index remained stable throughout the in-season period (Figure 3). This stability highlights the potential usefulness of the dRM-Index for guiding individualized strength and conditioning interventions. The dRM-Index during M−2, M−1 and matches showed *large* relationships with baseline neuromuscular performance (Figure 4). Slower players at 20 and 30 m reported a higher dRM-Index (“endurance-respiratory” profile; dRM-Index > 1), while faster players tended to report a lower dRM-Index (“speed-muscular” profile; dRM-Index < 1). Thus, a factor explaining the contrasting responses to the sessions between young male (Gil-Rey et al., 2015) and our female players might be related to the different locomotor profile (Garcia-Tabar et al., 2022) that accounts for different muscle fiber typology (Ansdell et al., 2020) and substrate utilization (Tarnopolsky et al., 1990), as previously explained. To note, the neuromuscular robust improvements found in these young female players (Table 1) are not usually observed in senior (Los Arcos et al., 2014) or young (Arregui-Martin et al., 2020; Gil-Rey et al., 2015) male players. The different locomotor profile (more oxidative for females than males for the same relative exercise intensity) previously described (Garcia-Tabar et al., 2022) agrees with the general dRM-Index (> 1, “endurance-respiratory”) found in this study, and suggests that in contrast to their male counterparts, young female players may exhibit a broader range of neuromuscular improvement during soccer activities. Hence, this study on dRM-Index applicability offers a novel and practical approach to individualize training of players to avoid no desired or “maladaptive” training responses and to optimize players’ preparedness to compete. Further studies are required to confirm the dRM-Index on-field utility for soccer.

While our study provides valuable insights, it is essential to acknowledge inherent limitations, a common aspect in research conducted within the dynamic context of competitive soccer teams. One notable limitation is the constrained sample size, a consequence of injured players at the time of testing sessions, the regular inclusion of some players in inferior or superior teams, and the exclusion of goalkeepers—factors that are common in soccer academies. To address this limitation, some studies have incorporated more than one team (Arregui-Martin et al., 2020; Garcia-Tabar et al., 2022). However, considering alternative teams from the same age group but different clubs was deemed unsuitable for this study. Different teams exhibit distinct weekly organization and training content, potentially introducing distortions in the results of TLs. These limitations, though inherent, are crucial contextual factors that should be considered when interpreting our findings and they warrant attention in future research endeavors within similar settings.

Furthermore, we acknowledge the broader structural challenges involved in conducting research in female sport. Compared to male cohorts, studies involving female athletes often face limited access to participants, smaller sample sizes, and reduced institutional support, which constrains both the generalizability and depth of applied sport science in this population (Elliott-Sale et al., 2021; Emmonds et al., 2019). Addressing these limitations through more inclusive research frameworks and coordinated efforts to increase the volume and visibility of female-specific data will be essential to improve the quality and applicability of future studies in women’s sport.

## Conclusions

To the best of our knowledge, this is the first study describing the ratio between respiratory and muscular perceived training loads (dRM-Index) and its associations with neuromuscular performance in young female soccer players. The main conclusions were that: (1) the consistently described TL pattern across the week suggested a deliberate effort in preparing for upcoming matches; (2) the RPEres-TL exceeded the RPEmus-TL during soccer-specific training sessions, indicating a potential need for specific training strategies in females; and (3) baseline neuromuscular performance predicted dRM-Index responses to training sessions and matches and might serve for training individualization. Further studies are warranted to validate the observed differences between the RPEres-TL and the RPEmus-TL and to corroborate the associations between the dRM-Index and neuromuscular performance in youth female soccer players. The findings provide actionable evidence for practitioners and contribute to addressing the current sex imbalance in the literature on training load monitoring.
